# Angle of approach to the superior rotator cuff of arthroscopic instruments depends on the acromial morphology: an experimental study in 3D printed human shoulders

**DOI:** 10.1186/s13018-019-1486-1

**Published:** 2019-12-12

**Authors:** Menduri Hoessly, Samy Bouaicha, Thorsten Jentzsch, Dominik C. Meyer

**Affiliations:** Department of Orthopaedics, Balgrist University Hospital, University of Zurich, Forchstrasse 340, CH 8008 Zurich, Switzerland

**Keywords:** Shoulder, Arthroscopy, Portal placement, Acromion

## Abstract

**Background:**

Portal placement is a key factor for the success of arthroscopic procedures, particularly in rotator cuff repair. We hypothesize that the acromial anatomy may strongly determine the position of the shoulder bony landmarks and limit the surgeon’s freedom to position the arthroscopic approaches in direction towards the acromion. The purpose of this study was to analyze the relation between different acromial shapes and the freedom of movement of arthroscopic instruments relative to the rotator cuff from standardized arthroscopic portals in a laboratory study on 3D shoulder models.

**Methods:**

3D models of shoulders with a broad range of different acromial shapes were printed using CT and MRI scans. Angles from the portals to defined points on the rotator cuff and the supraglenoid tubercle were measured. In conventional radiographs, the critical shoulder angle, the scapular body acromial angle, and the glenoid acromial angle were measured and compared with the measured angles to the rotator cuff.

**Results:**

There was a large variation of angles of approach of instruments to the rotator cuff between the seven shoulders for each portal. From the joint line portal and the posterior edge portal, the biggest angles were measured to the posterior cuff. From the intermediate portal, the angles were largest to the intermediate rotator cuff and from the anterior portals to the anterior cuff. To the supraglenoid tubercle, best access was from anterior. For all portals, there was a big correlation between the glenoid acromial angle and the scapular body acromial angle with the angles of approach to the tendon and especially to the supraglenoid tubercle.

**Conclusion:**

The access to the rotator cuff from almost every portal is influenced by the acromial shape. As hypothesized, a small (small GAA) and flat (big SBAA) acromion provide an easier approach to the rotator cuff from almost every portal. Therefore, it may severely influence the instruments maneuverability.

## Background

Portal placement is crucial in shoulder arthroscopy not only to protect the neurovascular structures but also for good visualization and range of motion of surgical devices, such as tendon perforators [1–5]. It appears advantageous to plan preoperatively which portals to use and what instruments to choose particularly in technically more demanding procedures such as rotator cuff repair [6]. However, we made the observation that in arthroscopic shoulder surgery, the subacromial space is not equally easy to access in all patients, even though the portals are usually placed in the same manner relative to the bony landmarks. We hypothesize that different shapes and orientations of the acromion as described in various anatomical studies [7–10] might be a determining factor for the freedom of instrument positioning and mobility. In order to handle and penetrate a torn rotator cuff for suture placement, the suture-passing instrument used should preferably pass the tendon in a sufficiently steep angle which also allows to integrate different layers of a delaminated tendon tear. If the angle of approach with an instrument such as tendon perforators is too small, the instrument may only scratch the surface and the point of entry and exit of the tendon and the direction of perforation is very hard to control.

However, to the best of our knowledge, no study has investigated the influence of the size and the shape of the acromion as the determining anatomical landmark on the range of motion of arthroscopic instruments in the subacromial space. We hypothesized that the acromial shape and orientation have a significant influence on where arthroscopic instruments such as tendon perforators may enter the subacromial space and consequently on their freedom of motion of there. In particular, we hypothesized that a steep and dorsally orientated acromion will reduce the possible angle of approach to the rotator cuff from the posterior portals, while a small acromion provides easy access to the rotator cuff from the majority of the portals. The purpose of this study was to analyze the relation between different acromial shapes and the freedom of movement of arthroscopic instruments relative to the rotator cuff. A further goal is to predict a possible difficulty on the range of motion on standard radiographs allowing to plan which portal should be used for which acromial shape when the superior rotator cuff is addressed.

## Methods

### Selection and 3D preparation of shoulders

Radiological studies of seven shoulders with a broad range of different acromial shapes were selected by two experienced shoulder surgeons. Three-dimensional MRI scans of three shoulders performed on a Siemens Avanto fit (1.5 T) scanner and three-dimensional CT scans of the other four shoulders performed on a Siemens Somatom Definition AS 64 scanner were selected. Of all shoulders, conventional X-ray with an anterior-posterior and Neer projection was available.

**Fig. 1:**
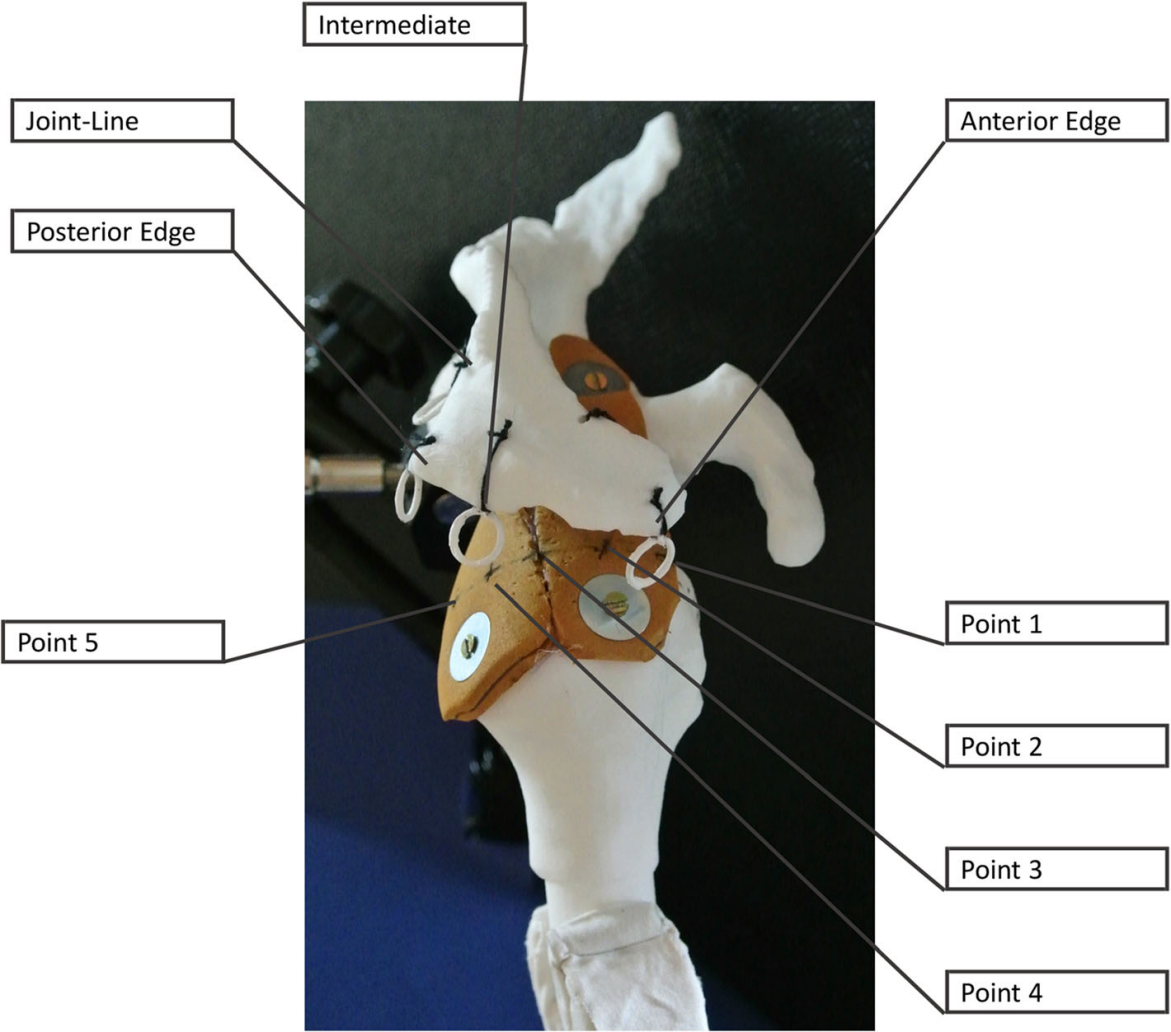
3D reconstruction of the shoulder with portals

All scans were anonymized and segmented by the first author using The Medical Imaging Interaction Toolkit (MITK, 2016.3, German Cancer Research Center, Heidelberg, Germany) [11]. The segmented data then were 3D printed with the Formiga Printer (EOS GmbH, Kraillingen, Germany). The material used was Polyamid 2200. The humerus and the scapula were independently printed and then immediately marked with the number 1–7. The humerus and the scapula were connected with a 5-mm elastic microcellular rubber as a simulation of the supraspinatus and infraspinatus tendons (Fig. 1). Their origin was attached in the supraspinatus and infraspinatus fossa, and the insertions were anchored at the greater tuberosity of the humerus.

### Radiographic measurements

**Fig. 2:**
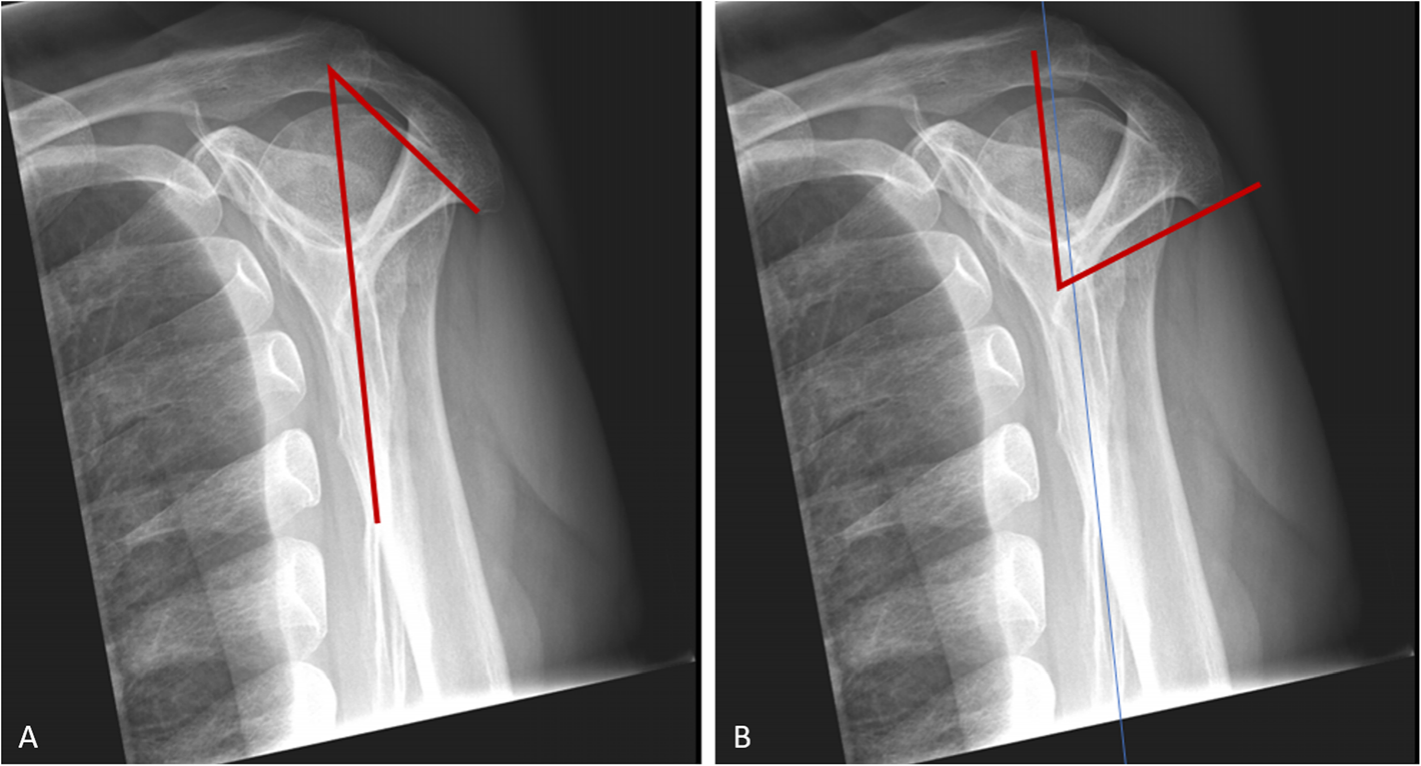
**a** Scapular body acromion angle (SBAA). **b** Glenoid acromial angle (GAA), determining the posterior extension of the acromion

The acromial morphology was defined by three values measurable on the standard radiograph of the shoulder. The critical shoulder angle (CSA) was measured according to Moor et al. [12]. The scapular body acromial angle (SBAA) was measured as described in Fig. 2a on the Neer projection. It is defined as an angle through the scapular body and the anteroposterior slope of the acromion. The third value used was the glenoid acromial angle (GAA) (Fig. 2b). This GAA is a value for the caudal orientation of the acromion and is measured on the Neer projection as described in Fig. 2b. It is defined as the angle between a line parallel to the scapular body through the middle of the glenoid fossa and a line from the most caudal edge of the acromion to the middle of the glenoid fossa. All radiological measurements were performed by the first and the senior author in consensus.

### Portal placement

Five typical arthroscopic shoulder portals were defined. All were placed along the lateral (and medial) border of the acromion in order to simulate the best possible access and therefore the largest possible angle of approach relative to the rotator cuff. One was placed at the extension of the glenoid surface (joint line), one at the posterior edge, one lateral of the acromion, and one at the anterior edge (Fig. 1).

### Angles and measurement

**Fig. 3:**
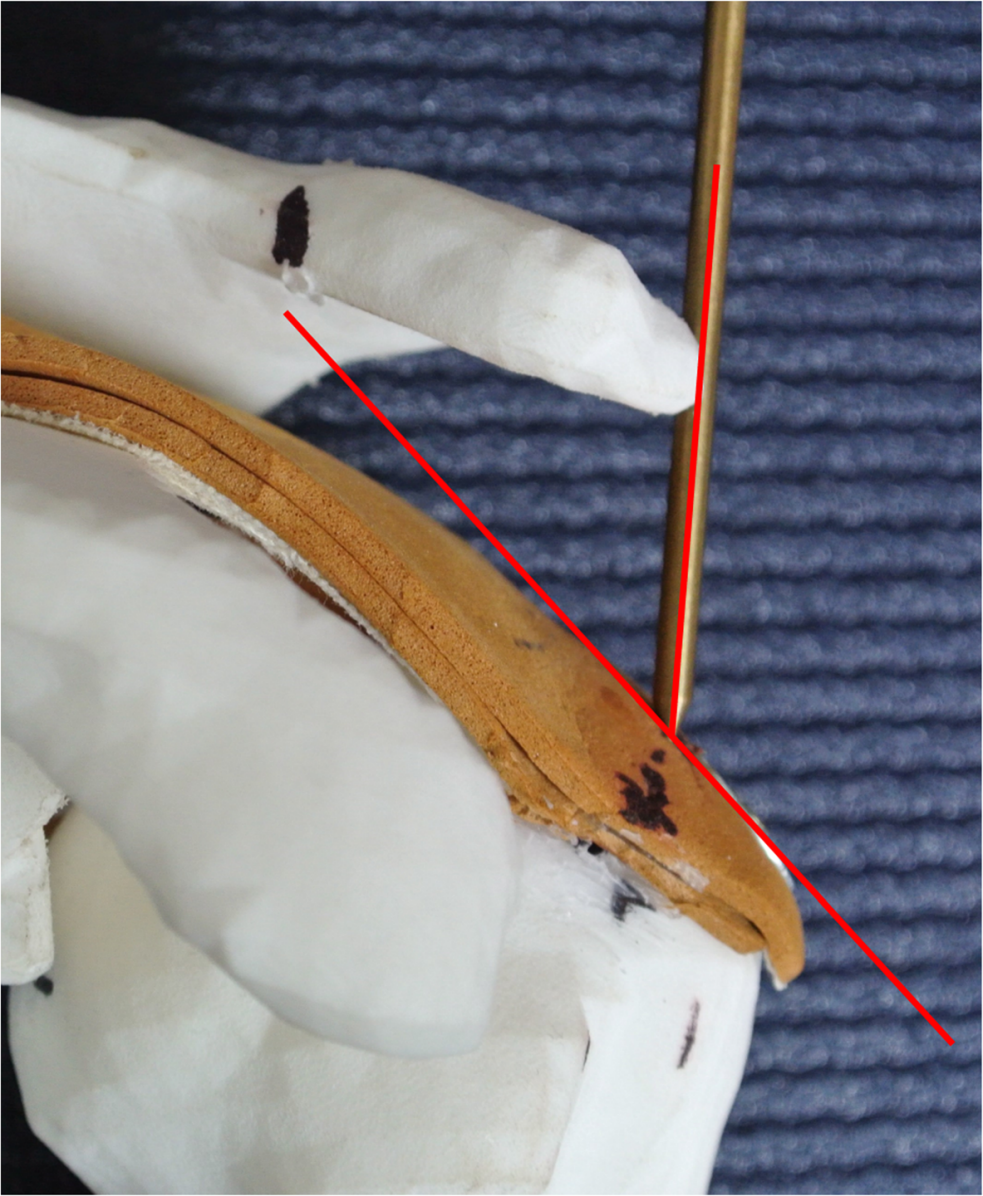
Angle measurement to the perforation points on the tendon

Angles of approach from the five portals to the tangent at 5 points on the rotator cuff tendon were measured. These points were chosen as typical sites of suture penetration occurring in cuff tear repair and were equally distributed on the medial edge of the cuff footprint on the greater tuberosity, between the bicipital groove and the posterior infraspinatus (Fig. 1). Using a pointed metallic rod of 3 mm diameter, each point was targeted from each portal on the acromion. To minimize perspective error, three pictures were taken for each measured angle of approach between the tendons and the axis of the rod (Fig. 3). The angles between the rod and the tangent of the tendon at the perforation points (“angles of approach”) were then measured using the ImageJ software [13]. The mean value of the three measurements was taken for statistical analysis. In a next step, angles from the portals to the line through the glenoid fossa and through the supraglenoid tubercle (as needed for anchor placement in SLAP repair) were measured using a goniometer.

### Position of the shoulder

For the measurements, the shoulders were fixed in a working platform specifically produced for this study. In this working platform “Walimex Pro Magic Arm,” the scapula and the humerus can be freely positioned in space (WalimexPro, Photo Walser GmbH und Co KG, Burgheim, Germany). To provide a typical position for shoulder arthroscopy, the joint was distracted in the direction of the humerus by 30% of the head diameter and the humerus was placed approximately in a 20° forward flexion and in an abduction of approximately 20° [4] relative to the scapula.

### Statistical analysis

Values are provided as means and standard deviation (SD) since the majority of data was normally distributed according to the Shapiro-Wilk test for normality. Box plots are used to illustrate angles of approach between each portal and defined points. Pearson correlation was used to study correlations between angles of approach to the perforation points and to the supraglenoid tubercle versus radiographic parameters such as glenoid acromial angle, critical shoulder angle, and scapular body acromial angle. Statistical analysis was carried out with Stata (version 13.1; StataCorp LLC, College Station, Texas, USA).

### Ethics

All patients signed an informed consent form for anonymized use of their MRI data at the Balgrist University Hospital in Zurich. This was approved by the local ethics committee (cantonal ethics committee Zurich 2016-00660).

## Results

### Angles of approach

**Fig. 4:**
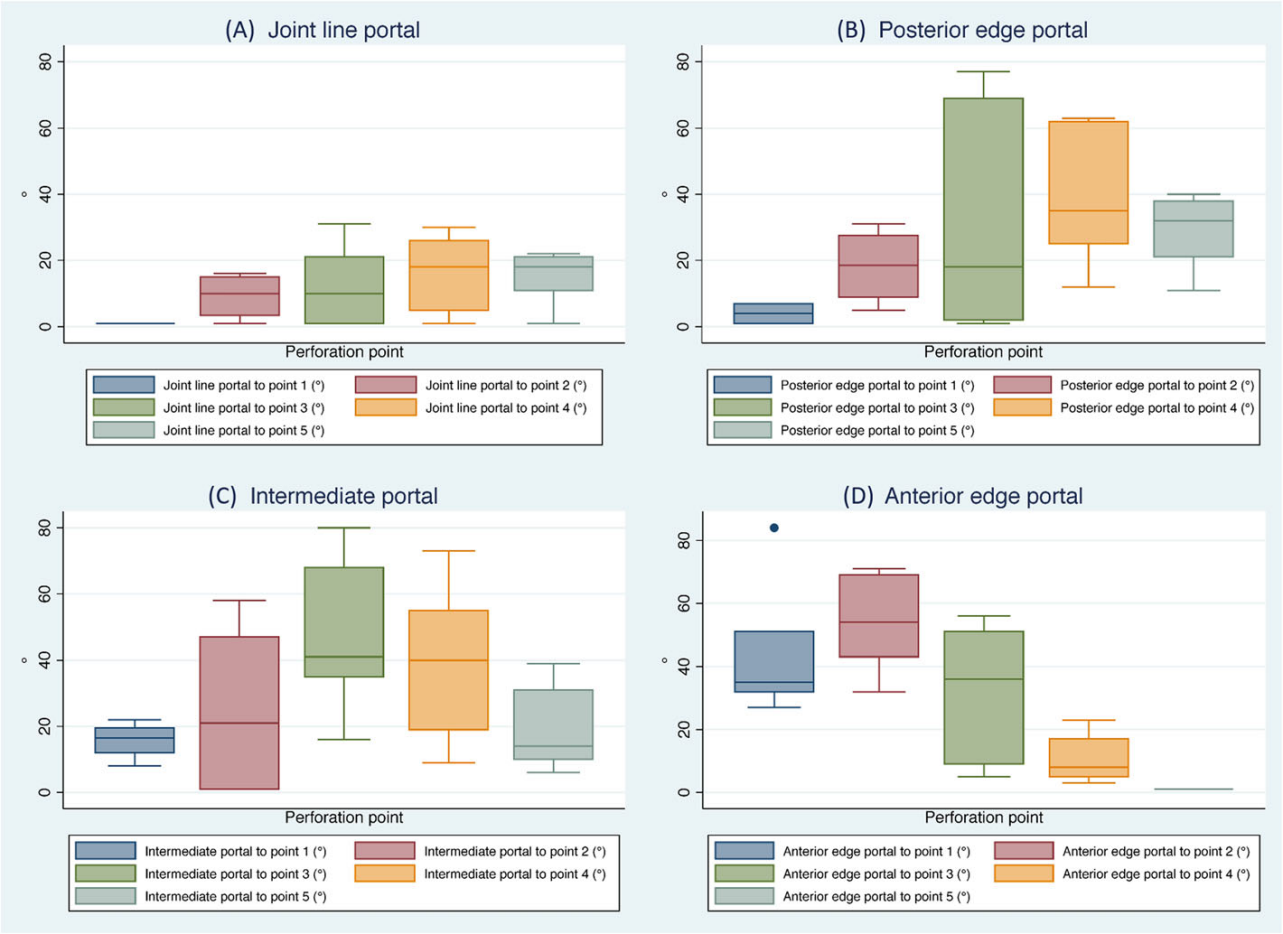
**a** Angles of approach (°) between the joint line portal and the perforation points 1–5 (Fig. 1). In none of the shoulders, a positive angle was obtained relative to the anterior edge of the supraspinatus tendon (perforation points 1). **b** Angles of approach (°) between the posterior edge portal and the perforation points 1–5. This portal showed the highest variability in relation to the posterior supraspinatus. **c** Angles of approach (°) between the intermediate portal and the perforation points 1–5. In 6 out of 7 of the shoulders through this portal, an angle of at least 10° could be obtained to the entire supraspinatus and infraspinatus tendons. Perforation points 1 and 2 were not reachable in 3 shoulders. **d** Angles of approach (°) between the anterior edge portal and the perforation points 1–5 (Fig. 1). In none of the shoulders, a positive angle could be obtained for the posterior edge of the infraspinatus (perforation point 5)

**Fig. 5:**
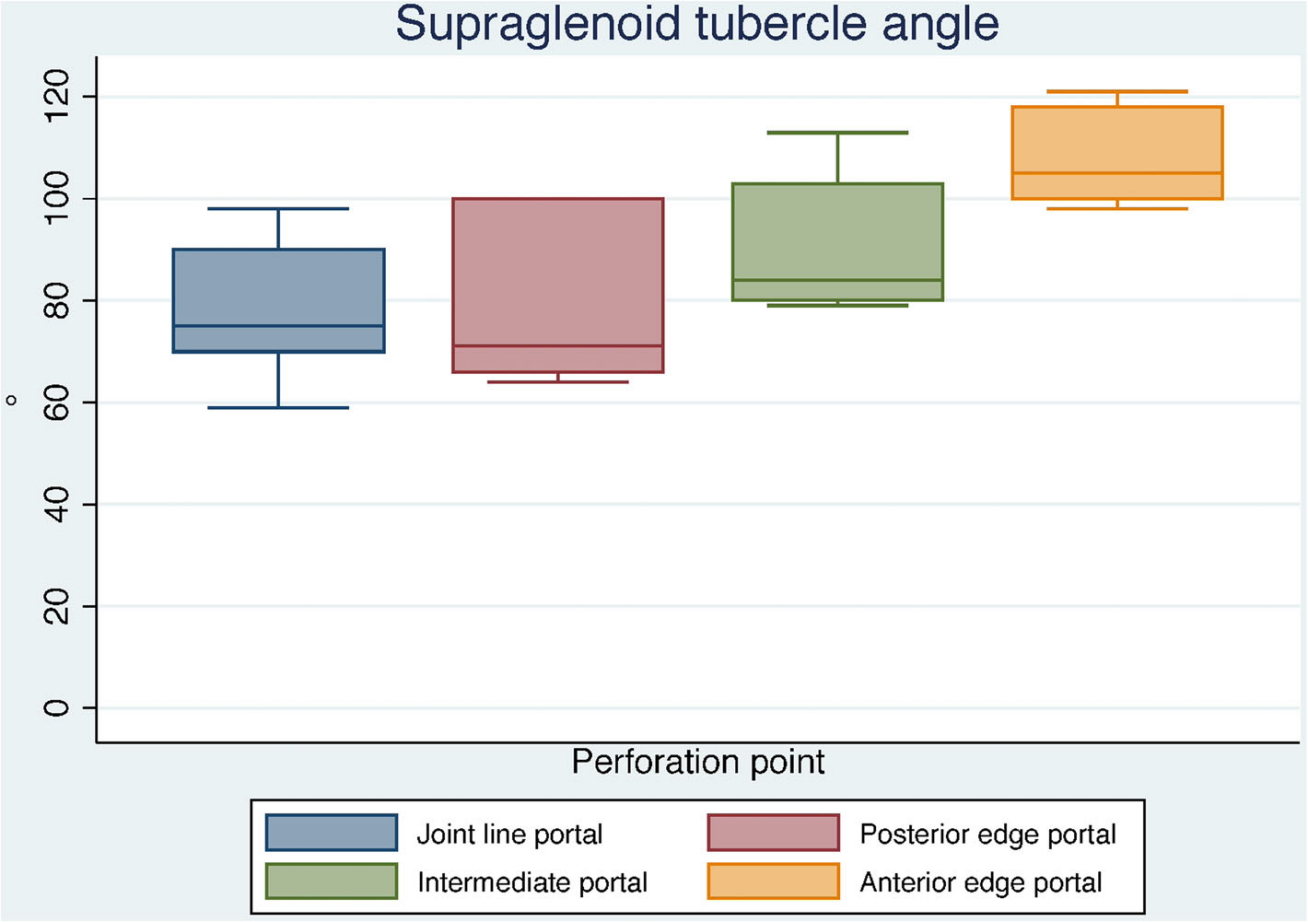
Angles of approach (°) to the supraglenoid tubercle from the arthroscopic portals in shoulders (*n* = 7). Larger angles were obtained from the anterior portals (98–121° vs. 59–98°)

In Figs. 4 and 5, the measured angles of approach between the different portals and the defined perforation points on the rotator cuff, and the angle to the supraglenoid tubercle are shown. The angles of approach for each portal show a large variation between the different shoulders. In Fig. 4a, the values for the joint line portal are indicated. From there, the most anterior point on the humerus was not reachable in any of the shoulders. The largest variation was found for perforation point 3 (posterior supraspinatus) and perforation point 4 (anterior infraspinatus). The biggest mean value was achieved for the posterior perforation points 4 and 5. Also, the angles to the supraglenoid tubercle are different for each shoulder. The correlation between the angles of approach from this portal and the acromial length (GAA) and slope (SBAA) was high, especially for the angle to the supraglenoid tubercle (GAA *R* = − 0.59; *p* = 0.16, SBAA *R* = 0.83; *p* = 0.02 [significantly different]).

In the more laterally placed posterior edge portal, the angles of approach to the perforation points were larger, as well as the variation of the angles of approach between the shoulders (Fig. 4b). The biggest variation was found in the angle of approach to the perforation point three (1–77°). Again, the largest mean values are found for the posterior perforation point 4, followed by points 3 and 5. For this portal, a high correlation was found for the angles of approach and the shape of the acromion. The biggest correlation with a *R* of − 0.73 (*p* = 0.061) for the GAA and a *R* of 0.75 (*p* = 0.052) for the SBAA was again found for the angles of approach to the supraglenoid tubercle. However, due to the large variety of factors, statistical significance was reached for none of the correlations.

From the intermediate portal angles of approach with large variation between the shoulders to perforation points 3 (16–80°) and 4 (9–73°) (Fig. 4c) were found. A high correlation between these angles and the GAA (*R* = − 0.57; *p* = 0.18) followed by the SBAA and the CSA with *R* of 0.45 (*p* = 0.31) each was found.

A different situation was found in the measurements from the anterior edge portal (Fig. 4d). There we found the biggest angle of approach to the most anterior perforation points 2 and 1 (69° and 84°) with a relevant influence of SBAA (*R* = 0.7; *p* = 0.09) and CSA (*R* = 0.6; *p* = 0.17).

### Morphological measurements in radiographs

The radiologically measured angles for the acromion resulted in a wide range between 46 and 80° for the GAA and a range between 38 and 76° for the SBAA. A lower range was shown for the CSA (29–37°).

## Discussion

Preoperative planning is an important factor for consistent quality of surgical procedures, which also applies to arthroscopy. The experimental results of this study confirm our hypothesis that conventional preoperative imaging may be of considerable help in the planning of arthroscopic portal placement. The acromial size and position determines the possible placement of portals and therefore the freedom of mobility that surgical instruments will find in the subacromial space. As expected, the anterior supraspinatus tendon was very difficult to reach from the most posterior (“joint line”) portal and vice versa the posterior infraspinatus difficult to reach from the most anterior (“anterior edge”) portal, independent from the acromial shape. The highest variability of the angles of approach that a surgical instrument will find relative to the tendon surface was at the posterior supraspinatus tendon (perforation point 3). This was most evident from the posterior edge portal with a range of 0–77°. Interestingly, from this portal, the posterior supraspinatus and entire infraspinatus were best accessible, also better than from the more posterior joint line portal. The most versatile access to the rotator cuff could be achieved through the intermediate (lateral) portal with positive angles of approach for the entire width of the supraspinatus and infraspinatus in most of the shoulders. If the anterior supraspinatus is to be addressed, the best mobility of the surgical instrument will be achieved through the anterior edge portal and to a lesser extent also through the intermediate portal.

**Fig. 6:**
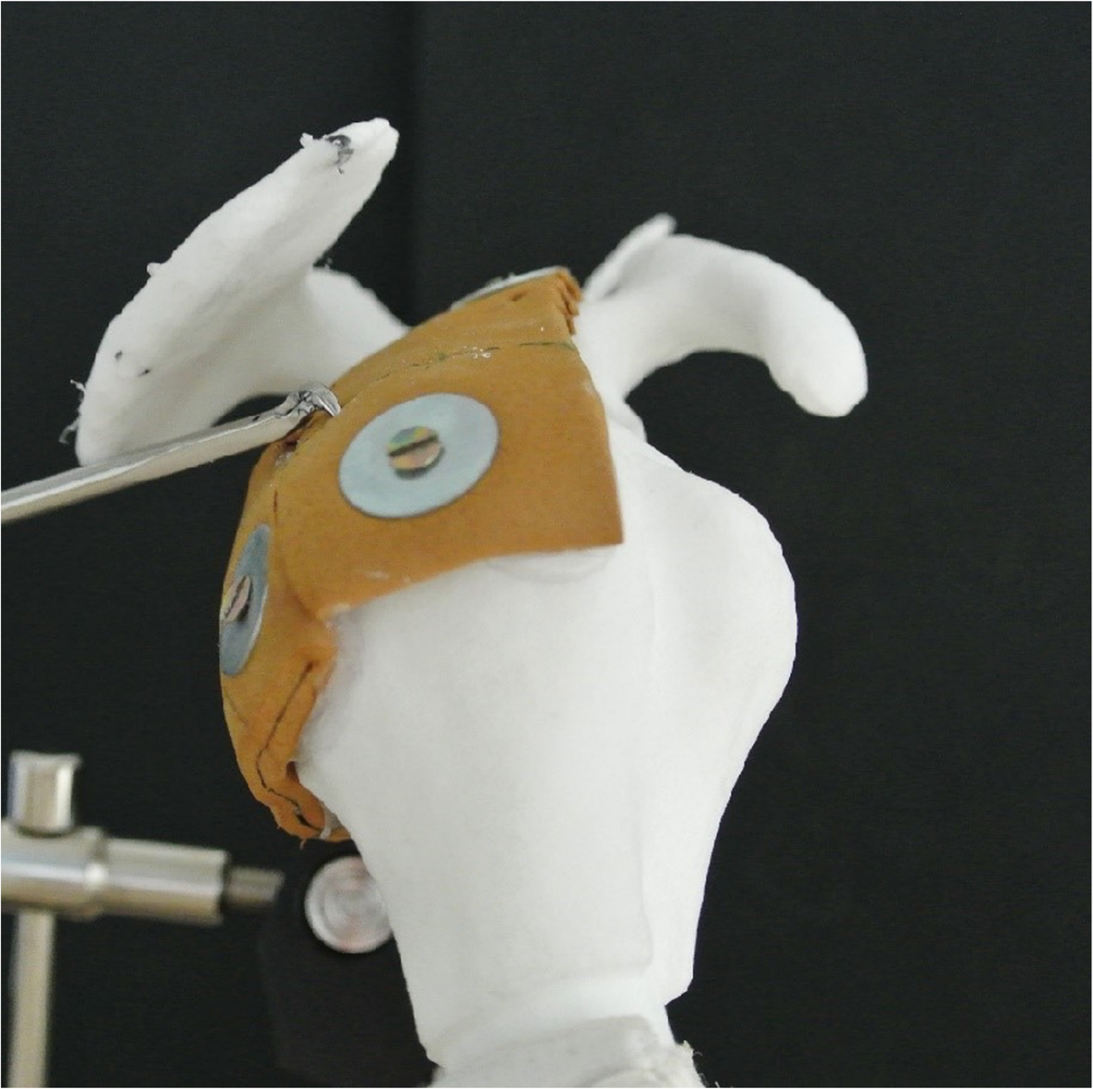
Shoulder with small angle of approach from the joint line portal

**Fig. 7:**
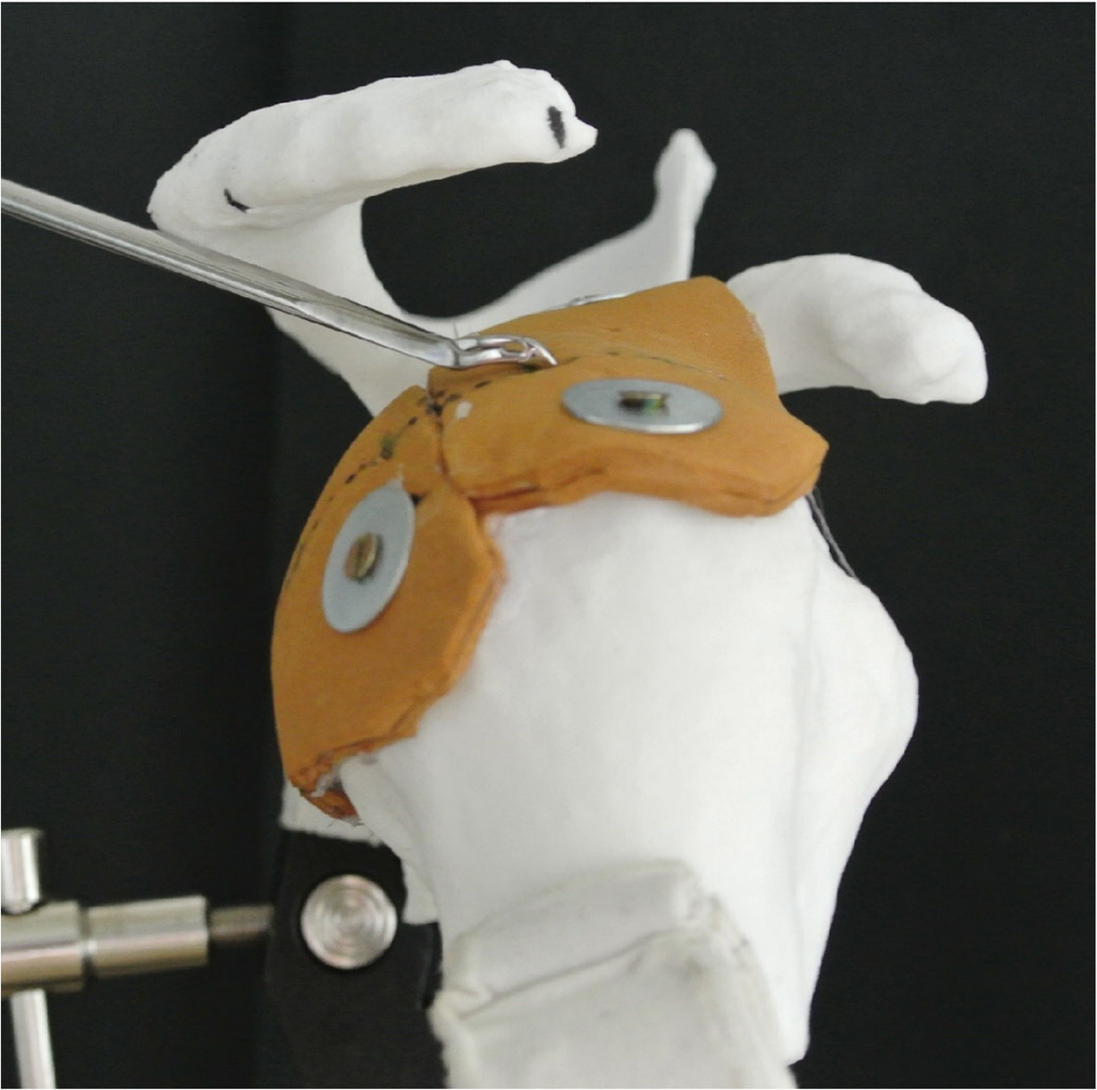
Shoulder with large angle of approach from the joint line portal

A new finding in this article is that the portal position and therefore subacromial maneuverability is strongly determined by the shape of the acromion. A large (big GAA) and a steep (small SBAA) acromion make the arthroscopy more difficult due to smaller angles of approach. However, a small and flat acromion, with small GAA and CSA and big SBAA allows for a larger angle of approach. This is especially the case for the joint line portal and the posterior edge portal as visualized in Figs. 6 and 7. For these two portals, the GAA and SBAA appear to be more important than the CSA. On the other hand, for the intermediate and anterior portal, the CSA appears to be the determining factor. This is not surprising, as the CSA is measured in the AP radiograph and is therefore a value for the lateral expansion of the acromion.

Baechler et al. described that 25° (corresponding to 115° supraglenoid tubercle angle) is desirable as a minimum arc of vertical clearance for arthroscopic instrumentation of the superior glenoid through lateral portals [14]. This requirement could be most reliably achieved through the anterior edge portal, relatively independent of the acromial shape (Fig. 5).

However, there are some limitations of this study. The most important confounding factor is that the surgeon has the intraoperative possibility to adapt the shoulder distraction, abduction, flexion, and rotation, which is an important factor to adapt to anatomical variations. Further, the possible combinations of the analyzed anatomical variables cannot be fully represented by the used samples. Other variables such as soft tissue coverage, tendon thickness, medial location of a possible tear, and mobility of the tendon could also not be reproduced in the used technique. It may be assumed that a large soft tissue coverage even potentiates the observed osseous restrictions on instrument maneuverability. Finally, the optimal angle of approach will also be determined by the type of surgical instrument used. Despite these limitations, however, the hypothesized relevance of anatomical variations of the acromion for the placement of arthroscopic portals and the consequent influence on surgical maneuverability could unambiguously be demonstrated and should be further assessed in the intraoperative clinical setting.

## Conclusion

This study shows for the first time that depending on the acromial shape, defined points on the rotator cuff may either be well within reach or inaccessible through the same portal. As hypothesized, a small (small GAA) and flat (big SBAA) acromion provide an easier approach to the rotator cuff from almost every portal, while a larger acromion results in a more challenging procedure, especially from the posterior portals. Analysis of the lateral (Neer) view gives an estimation for the posterior acromial extension and therefore for the position of posterior and posterolateral portals. This information may be useful for the careful planning of portals and instruments in arthroscopy.

## Data Availability

The datasets used and analyzed during the current study are available from the corresponding author on reasonable request.

## Abbreviations

CSA: Critical shoulder angle
GAA: Glenoid acromial angle
SBAA: Scapular body acromial angle
